# Copland’s Medical Dictionary

**Published:** 1845-06-01

**Authors:** 


COPLAND'S MEDICAL DICTIONARY is another English work somewhat analagous in its character and scope, which is now in progress of republication, by the Harpers of New-York, under the direction of Prof. C. A. Lee. Of this we are not, as yet, prepared to speak from personal examination.

